# A Silver Monochrome “Concetto spaziale” by Lucio Fontana: A Spectroscopic Non- and Micro-Invasive Investigation of Materials

**DOI:** 10.3390/molecules27144442

**Published:** 2022-07-11

**Authors:** Margherita Longoni, Carlotta Beccaria, Letizia Bonizzoni, Silvia Bruni

**Affiliations:** 1 Department of Chemistry, University of Milan, 20133 Milan, Italy; margherita.longoni@unimi.it; 2 Beccaria Carlotta & C. Studio Di Restauro, 20122 Milan, Italy; beccaria.restauro@gmail.com; 3 Department of Physics, University of Milan, 20133 Milan, Italy

**Keywords:** XRF, FTIR, Raman spectroscopy, SEM-EDX, FT-Raman, modern painting, metallic paint

## Abstract

In several of his artworks, for instance the *Venezie* cycle, Fontana employed metallic paints; previous investigations on such materials highlighted the use of different synthetic binders and of thick paint layers below the metal one, having different colours to change the visual perception of the metallic surface. In the present work, a monochrome silver “Concetto spaziale” by the Italo–Argentine artist belonging to a private collection recently gifted to the museum of the Church of San Fedele in Milano, Italy, was investigated to deepen the knowledge of this particular group of Fontana’s paintings. The artwork was initially visually inspected in visible and ultraviolet (UV) light. Subsequently, a non-invasive spectroscopic investigation was performed by X-ray fluorescence (XRF), reflection Fourier-transform infrared (FTIR) and Raman spectroscopy. A minute fragment of silver-coloured paint was taken from the reverse of the painting, near the cut edge, and examined by scanning electron microscopy coupled with energy dispersive X-ray analysis (SEM-EDX) and micro-Fourier-transform Raman (FT-Raman) spectroscopy. The analytical data made it possible to identify the composition of the metallic paint layer and of the underlying dark one, both from the point of view of the pigments and of the binders used, also highlighting the potential of the non-invasive and micro-invasive methods adopted in the investigation.

## 1. Introduction

The worldwide recognized importance of the pictorial works by Lucio Fontana, the well-known Italian–Argentine artist (1899–1968), derives both from the continuous research and innovation of his figurative and abstract way of expression, and from his pioneering use of innovative materials. These include, for example, fluorescent colours and many different synthetic paints, such as nitrocellulose, alkyd, polyvinyl acetate and acrylics, which became commercially available as he created his artworks and which he often used in multiple layers and together [1,2,3,4].

In the present work, a multi-technique approach was applied to investigate the materials in a painting by Lucio Fontana, preserved at the exhibition hall of the Church of San Fedele in Milano and belonging to Fontana’s *Tagli* (Cuts) series. The artwork, a “Concetto spaziale-Attesa” (Spatial Concept-Wait) dated 1961, is particularly interesting as it is a silver monochromatic painting and therefore belongs to those works in which the artist employed a metallic paint.

A few previous investigations have been published about the materials of Fontana’s works based on metallic paints. In the paintings of the *Venezie* cycle, even if considered to be part of the *Olii* series, the use of a synthetic alkyd resin as a binder has been identified [1,2,5]. The reason for this choice was traced to the considerable thickness of the paint layer which, if obtained with a drying oil, would probably show significant drying craqueleure. More precisely, in Fontana’s silver paintings, the thick paint layer usually lies under a very thin layer of silver-coloured paint and has different colours, ranging from ochre to red or black, to change the perception of the metal surface above [2]. Furthermore, pyrolysis–gas chromatography–mass spectrometry analyses showed, at least in one case (painting 61 O 41), the presence of low quantities of polyvinyl acetate (PVA) in addition to alkyd resin [1], while in another *Venezia* painting (61 O 53), a silver acrylic coating was detected on a hard polyester layer [6].

In the present work, characterization of painting materials was carried out to answer specific questions raised by conservation issues and restoration needs. It is not necessary to point out that the precise knowledge of materials is mandatory to perform correct and long-lasting restoration. A sequence from general to specific was adopted in the investigation. It is worth noting that scientific analyses, and in particular non-invasive procedures, are becoming more and more applied also in cases of modern and contemporary paintings [7,8,9] and to get information about degradation phenomena [10]. After a visual inspection in both visible (also with an optical digital microscope, OM) and ultraviolet light, we approached the problem using non-invasive techniques, in detail X-ray fluorescence (XRF), reflection Fourier-transform infrared (FTIR) and Raman spectroscopy. These techniques were chosen for their proven synergy on pictorial materials [11,12] and due to their different penetration depths, which can help reconstruct stratigraphic sequences without sampling, opening up interesting applications to off-limits masterpieces, such as the present case. Furthermore, all these techniques together present the ability to investigate different types of materials (organic, or inorganic such as vitreous or metallic). XRF is useful to get a rapid and reliable determination of medium-heavy elements, even when present in light matrix [13,14], without any sample preparation and regardless of the substrate material. This allows individuation of the pigments used in a wide variety of artefacts in a non-invasive way [15,16], even on modern materials [17]. XRF penetration depth depends on the matrix and the atomic number of the investigated element, and it can range from tens of micrometres to a few millimetres; the synergy with techniques with different penetration depths can give hints about the stratigraphy of the different layers of material [18]. Reflection FTIR spectroscopy can provide information on binders, even synthetic ones [19], adhesives [20] and inorganic materials such as carbonates or sulphates [21] with a penetration depth of about ten microns. Raman spectroscopy is suitable for the identification of pigments, both inorganic [22] and synthetic organic [23,24]. The penetration depth of the Raman technique depends on the excitation wavelength used and its absorption by the material examined, and can in principle be greater when using NIR exciting radiation [25]. In the present work, due to the physical properties and chemical composition of the metallic paint, more complete characterization of this material also required micro-analyses in addition to the non-invasive ones, carried out on a small sample taken from the back of the painting near the edge of the cut. To this end, scanning electron microscopy coupled with energy dispersive X-ray analysis (SEM-EDX) was used for elemental analysis, while micro-Fourier-transform Raman (FT-Raman) spectroscopy was exploited to identify the binder.

## 2. Materials and Methods

### 2.1. The Painting

The work, preserved in the exhibition hall of the Church of San Fedele in Milano (as indicated above), was created on a canvas measuring 99.8 × 79.8 cm. It bears the artist’s signature and a numerical sequence (1 + 1-7741F) on the back.

Belonging to the *Nanda Vigo* collection, this work was created by Fontana using silver-coloured paint and is characterized by a diagonal texture of considerable thickness, probably made with the aid of a paintbrush.

**Figure 1:**
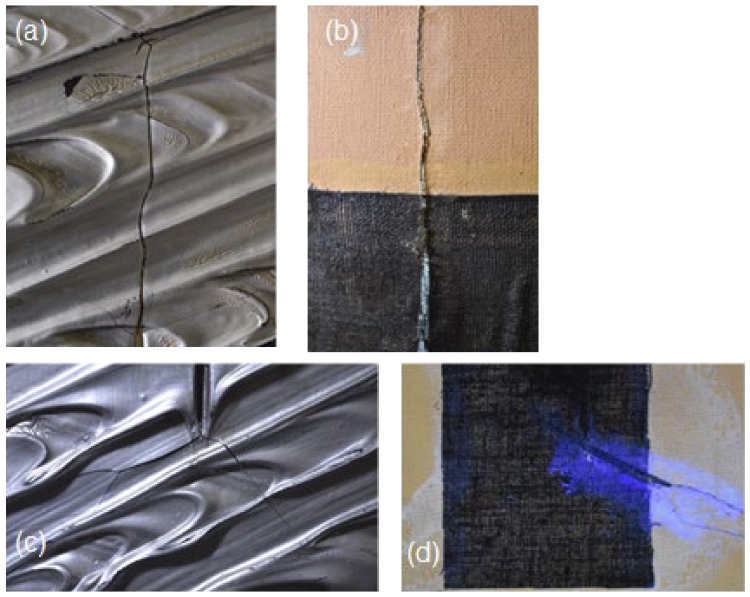
Some details of alterations observed in the monochrome silver “Concetto spaziale” by Lucio Fontana: (**a**) crack developed from the upper end of the cut; (**b**) tear in the original canvas and “teletta”; (**c**) degradation of the pictorial material; (**d**) old restoration in the lower area of the cut, with presence of a textile patch, observed under UV light.

The canvas showed deformations caused by the considerable layer of colour spread on the front, as well as an extension of the original cut in the upper part (about 10 cm) where the threads of the original canvas had broken. The cut was “closed” on the back by the “teletta”; this had been placed by the artist, but the painting suffered structural damage in the area of the tear, where it was detached (Figure 1).

An old restoration intervention was visible on the lower side of the cut; in fact a patch, also black, had been placed over the “teletta”.

A previous restoration intervention was also visible due to the presence of altered retouchings carried out in the upper left and right corners of the painting.

The front of the work was affected by an aesthetic deterioration caused during the drying of the material (Figure 1); yellowed areas could be seen in the thickest and most depressed spots due to accumulation of binder.

Some small micro-holes of the silver layer suggested the presence of an underlying black layer. This led to the supposition that the silver layer was only a surface finish, as reported by some of the texts examined [2].

The artist’s cut, placed in the central part of the painting, showed slight deformation, and its edges fell toward the “teletta”.

The work was placed inside a wooden frame with plexiglass protection.

Restoration was also performed on the basis of the scientific analyses. After the surface was dusted with soft brushes, the “teletta” was detached and repaired, the interruption of the support canvas was restored, the structure was reinforced from the back by strips of synthetic veil with Beva^®^ Gel as an adhesive, and then the “teletta” was repositioned. Aesthetic intervention was limited to lightening the visual interruption points of the monochrome drafting in order to restore the continuity and correct reading of the work. Finally, the work was placed back in the frame, and on the back, a cardboard plume panel was added to protect the work from weather variations and atmospheric dust.

### 2.2. Reference Materials

Alkyd and poly(vinyl)acetate binders were purchased from Kremer Pigmente (Aichstetten, Germany) to acquire reference spectra.

### 2.3. Measurement Areas

The different spectroscopic techniques listed above were applied to several areas of the silver-coloured surface, including some points where surface inhomogeneity was present, such as areas with pictorial layer leaks or binder stains, and an area with evident restoration. The reverse of the work was also considered, focusing in particular on the canvas and on the silver-coloured dripping near the edges of the cut.

The measurement areas on which XRF, reflectance FTIR and Raman analyses were performed are listed in Table 1. OM was also performed on some measurement areas, as indicated in the same table.

As mentioned in the Introduction, a small sample of silver-coloured paint taken from the back side of the painting near the edge of the cut (near Point 8) was also examined with FT-Raman and SEM-EDX.

### 2.4. Optical Microscopy

A portable digital optical microscope (DinoLite, 5Mpx) was used to get images with 50× or 200× magnification to support chemical analyses. The microscope was equipped with a polarising filter to reduce the gloss of the shiny surface.

### 2.5. X-ray Fluorescence (XRF)

XRF investigation was performed using an Assing LITHOS 3000 portable spectrometer, with quasi-monochromatic excitation at 17.4 keV (100 μm transmission Zr filter on a Mo target X-ray tube); the analysed area on the sample was about 4 mm radius. Working conditions were 25 kV and 300 μA, and the measuring time was 100 s (live time); the energy efficiency of the spectrometer is low for energies below 2.5 KeV. Sensitivity of the spectrometer is low for energy below 2.5 keV, not allowing the detection of light elements (from approximately Z = 17).

### 2.6. Reflection FTIR Spectroscopy

A Bruker Alpha FTIR spectrophotometer in reflection mode was used for non-invasive analyses. The instrument is equipped with a reflection module for contactless measurements and a deuterated triglycine sulphate (DTGS) detector, which operates at room temperature and guarantees a linear response in the spectral range between 7500 and 375 cm^−1^. An integrated camera of approximately 6 mm diameter allows the operator to select the area to be measured. FTIR spectra were acquired with a resolution of 4 cm^−1^ as a sum of 100 scans after the acquisition of the background spectrum on a gold mirror. The reflection spectra in the mid-IR (MIR) region were processed by Kramers–Kronig transform using Bruker OPUS software.

### 2.7. Raman Spectroscopy

A Bruker BRAVO handheld spectrometer was used for Raman measurements. This instrument is based on patented SSE™ technology, which provides the excitation of spectra by means of two diode lasers operating at different temperatures and emitting, respectively, at 785 and 850 nm. An appropriate algorithm allows extraction of the final Raman spectral data. The spectra are collected in two sequential steps, from 300 cm^−1^ to 2000 cm^−1^ and from 2000 cm^−1^ to 3200 cm^−1^. The average spectral resolution is approximately 11 cm^−1^. The applied laser power is less than 100 mW for both lasers, and the beam is focused on an area of approximately 500 μm × 100 μm, so the power density is really limited. The acquisition time and the number of accumulations were automatically set by the instrument. In particular, the Raman spectrum on Area 1 was recorded with an exposure time of 13 s and 2 accumulations, while the spectrum on Area 5 was recorded with an exposure time of 5.4 s and 8 accumulations.

### 2.8. FT-Raman Spectroscopy

FT-Raman spectra were recorded between 4000 and 200 cm^−1^ directly on the sample taken from the back of the painting, without any preparation. A Jasco FT-Raman RFT-600 spectrometer was employed, using the 1064 nm emission of a Nd:YAG laser for excitation. The laser output power was about 180 mW. Spectra were acquired as the sum of 300 scans. The resolution was 4 cm^−1^.

### 2.9. SEM-EDX Analysis

SEM-EDX analyses were performed on the same sample from the back of the painting using a Hitachi TM 1000 microscope with a resolution of 1 nm and equipped with an energy dispersion X-ray (EDX) spectrometer. The accelerating voltage was 15 kV.

## 3. Results and Discussion

Due to the synergy of the applied techniques, which exploited different penetration depths, and due to the presence of small gaps in the paint layers, it was possible to investigate the whole stratigraphy, composed of the preparation layer of the canvas, a dark layer and the silver-coloured paint.

### 3.1. Front of the Painting

**Figure 2:**
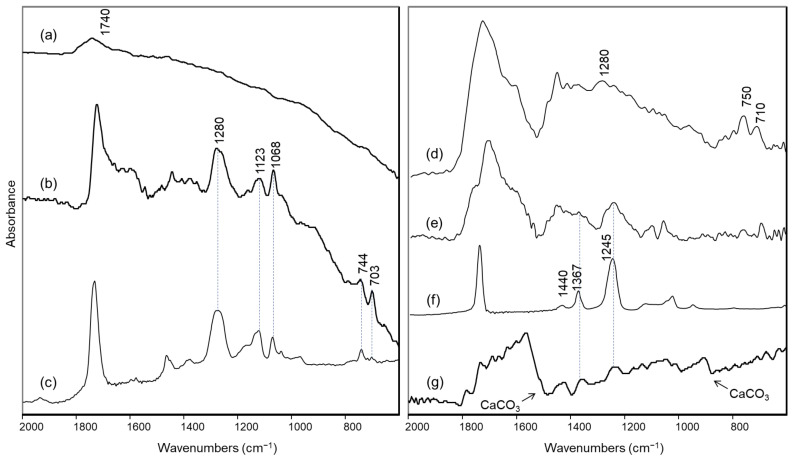
FTIR specular reflection spectra of areas on the front of the “Concetto spaziale”: (**a**) silver paint (Area 1); (**b**) black spot in an empty space of the silver paint (Area 2); (**c**) reference alkyd resin spread on canvas; (**d**) yellowish spot corresponding to an accumulation of binder (Area 3); (**e**) differential spectrum between spectra (**b**,**d**); (**f**) reference polyvinyl acetate spread on canvas; (**g**) possible retouching in the upper left corner of the painting (Area 4). In all cases the spectra shown were obtained from the experimental reflectance data using the Kramers–Kronig transform, with the exception of spectrum (**d**), which was obtained from a transparent material superimposed on the metallic surface, and is thus shown as pseudo-absorbance, log(1/R). In spectrum (**g**), the bands due to calcium carbonate are distorted due to specular reflectance of a material with a higher particle size.

On the front side of the artwork, the silver-coloured paint (Area 1) returned FTIR reflectance spectra with a characteristic slope towards lower wavenumbers due to the optical properties of the metallic surface (Figure 2a). In the same spectra, the only bands attributable to the binder were found around 2975, 2940 and 1740 cm^−1^ and did not allow its precise identification. At the same time, no relevant signals could be observed in the Raman spectrum.

In the small black areas observed in some empty spaces of the silver-coloured paint (Area 2), the presence of an alkyd component could be recognized, as demonstrated by the characteristic FTIR bands at 1280, 1123, 1068, 744 and 703 cm^−1^ (Figure 2b) [26], which are clearly evident in the reflection spectrum of an alkyd binder spread on canvas acquired as reference (Figure 2c). As its bands were detected in the gaps of the metallic paint, the alkyd binder is more reasonably associated with the underlying black paint layer.

For this reason, to acquire possible information about the binder in the silver-coloured paint, a yellowish stain present on the front of the painting (Area 3) and presumably due to accumulation of binder was examined by reflection FTIR spectroscopy. Even if the spectrum obtained was not of excellent quality, it still allowed us to observe once again the main bands due to an alkyd resin (Figure 2d), with the doublet around 700 cm^−1^ slightly displaced, probably due to distortion associated with the reflection conditions. However, in the region of the spectrum from 1250 to 1000 cm^−1^, different components seem to overlap. In order to possibly highlight these components, we decided to subtract the FTIR spectrum of the alkyd resin from that of the yellow stain. In the difference spectrum (Figure 2e), a band around 1245 cm^−1^ can now be clearly detected and, together with the signal around 1370 cm^−1^, can be possibly assigned to polyvinyl acetate (PVA) [20,27], as also confirmed by the comparison with the reference spectrum acquired for this binder (Figure 2f).

If, as suggested by Gottschaller for other paintings with silver-coloured paint by the same artist [2], the silver-coloured layer is very thin and superimposed on a sort of underlying “bole”, it can be hypothesized that the alkyd binder is due to the latter, while the vinyl binder is associated with the metallic paint. On the portion of silver-coloured paint in the micro-sample taken from the cut on the back of the work, it was in fact possible to acquire an FT-Raman spectrum with bands at 2939, 1734, 1438 and 632 cm^−1^ (Figure S1), attributable to polyvinyl acetate [27], but in that case it could also be due to the adhesive used to fix the “teletta” (see Section 3.2). Nevertheless, it should be emphasized that neither in the IR spectra nor in the Raman spectra obtained on the silver-coloured paint have ever been observed characteristic signals of an acrylic binder for this painting. In particular, the typical band of such a binder around 1170 cm^−1^ [19,20] is lacking in the IR spectra (Figure 2a,d), and the Raman bands at about 800–840 cm^−1^ reported in the literature for an acrylic polymer [28,29] were also not observed in the FT-Raman spectrum of the silver paint in the micro-sample (Figure S1).

**Figure 3:**
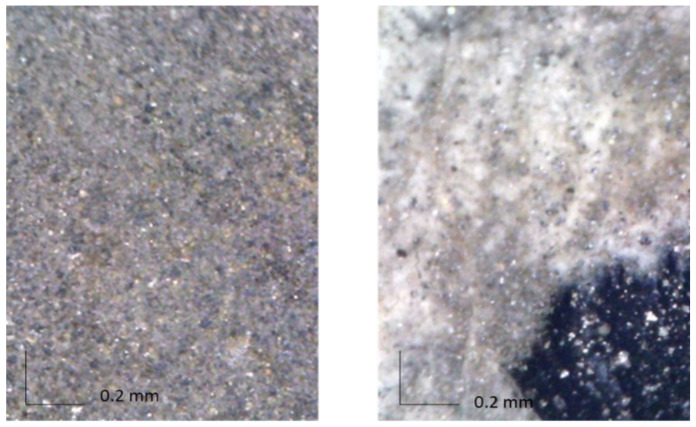
(**left**) OM (200×) of the front of the painting: small metallic grains and blue pigment grain are visible; (**right**) OM (200×) of the restored area on the front of the painting: the different aspect of the silver area is evident, together with the green painting.

As for the pigments, considering the XRF results summarised in Table 2, the comparison between the spectra obtained on the silver-coloured area (Point 1) and the gap of paint do not show any difference in the detected elements, but the change in the Compton scattering peak intensity indicates a higher presence of light elements in the silver-coloured paint. The same applies to Point 3, the yellow binder stain. A difference is instead evident if comparing the spectra obtained on the silver-coloured area on the front with those acquired on the back of the painting: in the front, the presence of Ba and Co is highlighted. The former, typical of modern painting, is in the form of barium sulphate or lithopone (if mixed with zinc sulphide), often used as filler or extender due to its stability. Possibly the painter used it to disperse silver-coloured material (see also MO image in Figure 3). Co is instead usually linked to the use of blue pigments; we can thus speculate (also on the basis of the MO images in Figure 3) the presence of small quantities of cobalt blue, which could also be the matter of the dark priming used as a ground. Unfortunately, no further evidence could be obtained for this pigment from the non-invasively acquired vibrational spectra, and no samples from the front of the painting were available for microanalysis in the laboratory. Anyway, even if the presence of Co alone could indicate a possible use of smalt blue, with the painting being a contemporary one, this pigment can be correctly excluded. Indeed, modern cobalt acrylic paints have been recognized in other Fontana works by Gottschaller et al. [1] and Ferriani et al. [3]. In particular, the latter paper reports the use of cobalt blue (acrylic).

**Figure 4:**
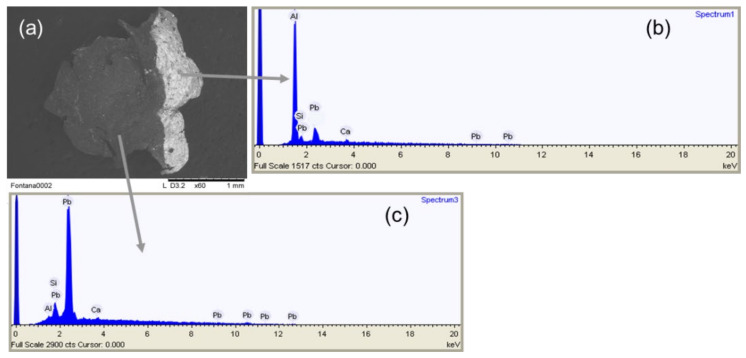
(**a**) SEM image of the sample taken from the silver dripping near the cut on the back of the “Concetto spaziale”; (**b**) EDX spectrum of the sample area with the highest mean Z value, corresponding to the silver paint; (**c**) EDX spectrum of the sample area with the lowest mean Z value, corresponding to preparation layer of the canvas.

The material responsible for the metallic appearance of the paint could definitely be recognised as aluminium thanks to SEM-EDX analysis of the small fragment taken from the edge of the cut on the back of the painting (Figure 4). It is interesting to note that in the part of the fragment not covered by the silver paint, only lead could be observed, in agreement with the results of non-invasive XRF analysis.

**Figure 5:**
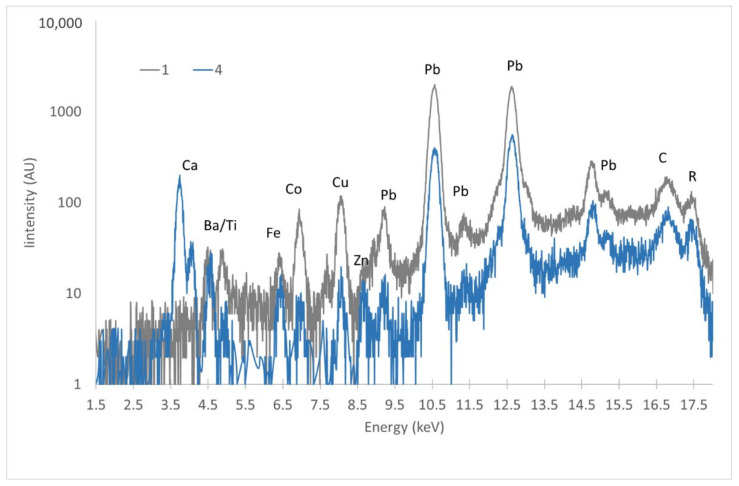
Comparison of XRF spectra in logarithmic scale for the original (1) and restored (4) areas on the front of the painting.

Finally, possible retouching has been identified in the upper left corner of the painting. The FTIR spectrum obtained there clearly shows the presence of calcium carbonate, again with a vinyl binder (Figure 2g). XRF as well reveals a high presence of Ca, together with Ti and Zn, while the Ba signal completely disappears (Figure 5).

### 3.2. Back of the Painting

The back of the canvas, uniformly beige in colour, merely shows the presence of lead (from white lead, as identified by reflectance FTIR, see below), for which both L series (9–15 keV) and M series (2.3–2.4 keV) are detected in XRF spectra. We can thus claim that the light brown/yellow shade is not given by the presence of heart/ochre. The detection of M lines indicates that lead is also present in the surface layer of the back of the canvas; these signals disappear when investigating the silver-coloured dripping, as even its small thickness can absorb such low-energy signals. On the other hand, in the dripping spectrum, the Compton scattering peak is more intense, indicating a lower average Z of the material, and thus the presence of non-detectable elements, such as Al, S and/or Cl, besides organic compounds. In this same spectrum, low signals of iron and copper are also present, correlated to the silver-coloured pigment, mainly composed of light elements, as shown by SEM-EDX (see Section 3.1). Indeed, very low signals due to traces of barium and cobalt are also detectable in this spectrum.

**Figure 6:**
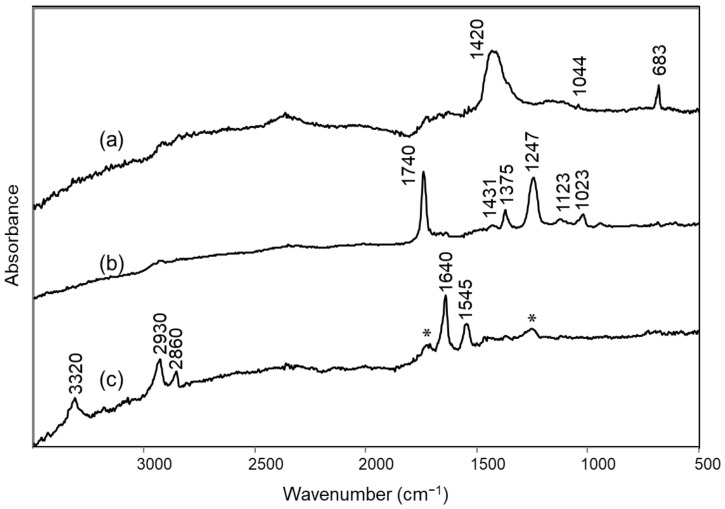
FTIR specular reflection spectra (after Kramers–Kronig transformation) of areas on the back of the “Concetto spaziale”: (**a**) beige canvas (Area 5); (**b**) adhesive residue that gives yellowish fluorescence in UV light (Area 6); (**c**) adhesive residue which gives white fluorescence in UV light (Area 7). In spectrum (**c**), the bands marked with an asterisk (*) are probably due to residues of the PVA adhesive.

Reflectance FTIR measurements were also performed on the back of the canvas (Area 5) and showed bands at 1420, 1044 and 683 cm^−1^ typical of lead white (Figure 6a). The Raman spectrum obtained on a corresponding area was also characterized by a sharp peak at 1050 cm^−1^ due to the same pigment (Figure S2). No traces of the components of so-called cementite, used in the preparation of the reverse of many of Fontana’s paintings [1] and usually containing calcium carbonate, could be detected by such techniques. In the literature, the use of lead white is mentioned for the preparation layer of another “Concetto spaziale” (60 O 81) [3] belonging to the *Olii* series, but has not been reported yet as primer for the reverse of Fontana’s painting. The only similarity with cementite-based preparations is the high density of the pigment [1], which is demonstrated by the fact that the bands due to lead white dominate the FTIR spectrum (also in the near IR region, data not shown), with the exception of a weak signal around 1730 cm^−1^, making it difficult to identify the binder used. Anyway, the creamy colour observed for the layer is most likely due to the ageing of the binder itself.

Still on the reverse of the painting, near the black gauze “teletta”, two different adhesives could be identified based on FTIR reflectance spectra (Areas 6 and 7). The first one, which produced a yellow fluorescence under UV light, was, as expected, a PVA-based material, most likely Vinavil^®^, commonly used by the artist to glue the “teletta” on the reverse of the cuts [2]. It was easily identified thanks to the bands at 1740, 1431, 1375, 1247, 1123 and 1023 cm^−1^ [27] (Figure 6b). The second, which produced white emission when irradiated with UV light (Figure 1d), was instead recognized as a polyamide adhesive based on its bands at 3320, 2930, 2860, 1640 and 1545 cm^−1^ [30] (Figure 6c), and was presumably used in the previous restoration on the underside of the cut.

## 4. Conclusions

Lucio Fontana was an indefatigable experimenter who borrowed the materials he used in his masterpieces from other fields, even from industrial experimentation. This attitude results, nowadays, in restoration challenges that cannot be faced without the help of scientific investigations to give information about materials and techniques. The present work takes a key piece in the bare literature about the subject and confirms the fundamental importance of scientific investigations prior to restoration for modern and contemporary art where artists experimented with new materials to express a new function of art.

Indeed, the analytical data obtained made it possible to identify the composition of the metallic paint and of the underlying dark layer, both from the point of view of the pigments and of the binders used, also highlighting the potential of the non-invasive and micro-invasive methods. The coupling of techniques with different penetration depths and the presence of some small micro-lacunae of the silver-coloured layer let us understand the stratigraphy of the painting, where the silvered layer is only a surface finish, as reported in other cases [2]. Moreover, two different types of binder for the silver and black colours were detected, confirming this hypothesis. In particular, in the silver-coloured paint, aluminium could be recognized as a metal, and poly(vinyl)acetate was hypothesized as a binder, while for the underlying black paint layer, an alkyd resin could be identified as a binder, and the presence of cobalt suggested the use of cobalt blue as a pigment. Finally, on the back of the painting, two different adhesives were recognized, namely one based on PVA, commonly used by the artist to fix the “teletta”, and a polyamide possibly employed in a previous restoration.

Results were thus used for the critical restoration performed, in particular for detachment of the “teletta” and for mending the tear. Assays and solubility tests were also performed based on the analytical results to decide which solvent to apply for the removal of the black canvas and the subsequent suturing of the interrupted threads. Acetone in a 10% and 20% thickened form in Nevek was used, with progressively longer setting times, in line with the vinyl nature of the adhesive used in laying the black cloth, as shown by the results of the scientific analyses. Thanks to this solution, it was then possible to detach the upper part of the canvas from the back of the cut by making the minimal amounts of solvent necessary for the reactivation of the glue, proceeding in parallel with a dry method for the mechanical removal of the adhesion at the points of greatest fragility of the fabric.

## Figures and Tables

**Table 1:** Areas of Lucio Fontana’s monochrome silver painting on which in situ spectroscopic measurements were performed.

Measurement Area	Techniques	Description
1 *	reflectance FTIR, XRF, Raman	front side, silver-coloured paint
2 *	reflectance FTIR, XRF	front side, black spot (gap in the silver-coloured paint)
3 *	reflectance FTIR, XRF	front side, yellow stain
4 *	reflectance FTIR, XRF	front side, upper left corner, possible retouching
5 *	reflectance FTIR, XRF, Raman	back side, canvas
6	reflectance FTIR	back side, adhesive residue
7	reflectance FTIR	back side, adhesive residue
8 *	XRF	back side, silver paint dripping

* These measurement areas were also subjected to optical digital microscope (OM).

**Table 2:** Elements detected by XRF on the areas described in Table 1, with the exception of Area 8, which is extensively discussed in the text.

Measurement Area	Principal Elements	Trace Elements
1 *	Pb	Cu, Co, Ba (Zn)
2 *	Pb	Cu, Co, Ba
3 *	Pb	Cu, Co, Ba
4 *	Ca, Pb	Ti, Fe, Cu
5 *	Pb	

* These measurement areas were also subjected to optical digital microscope (OM).

## Data Availability

Not applicable.

## Supplementary Materials

The following supporting information can be downloaded at https://www.mdpi.com/article/10.3390/molecules27144442/s1. Figure S1: FT-Raman spectrum of the silver paint in the sample taken from the dripping on the back of the canvas; Figure S2: Raman spectrum from the back of the canvas.

## Sample Availability

Not available.
