# Supplementary material for: A Silver Monochrome “Concetto spaziale” by Lucio Fontana: A Spectroscopic Non- and Micro-Invasive Investigation of Materials
Source: Molecules. 2022 Jul 11;27(14):4442. doi: 10.3390/molecules27144442 (PMC9320160; doi:10.3390/molecules27144442)
Supplement: Supplementary file 1 [file molecules-27-04442-s001.zip › molecules-1773810-Supplementary.pdf]

Supplementary Materials

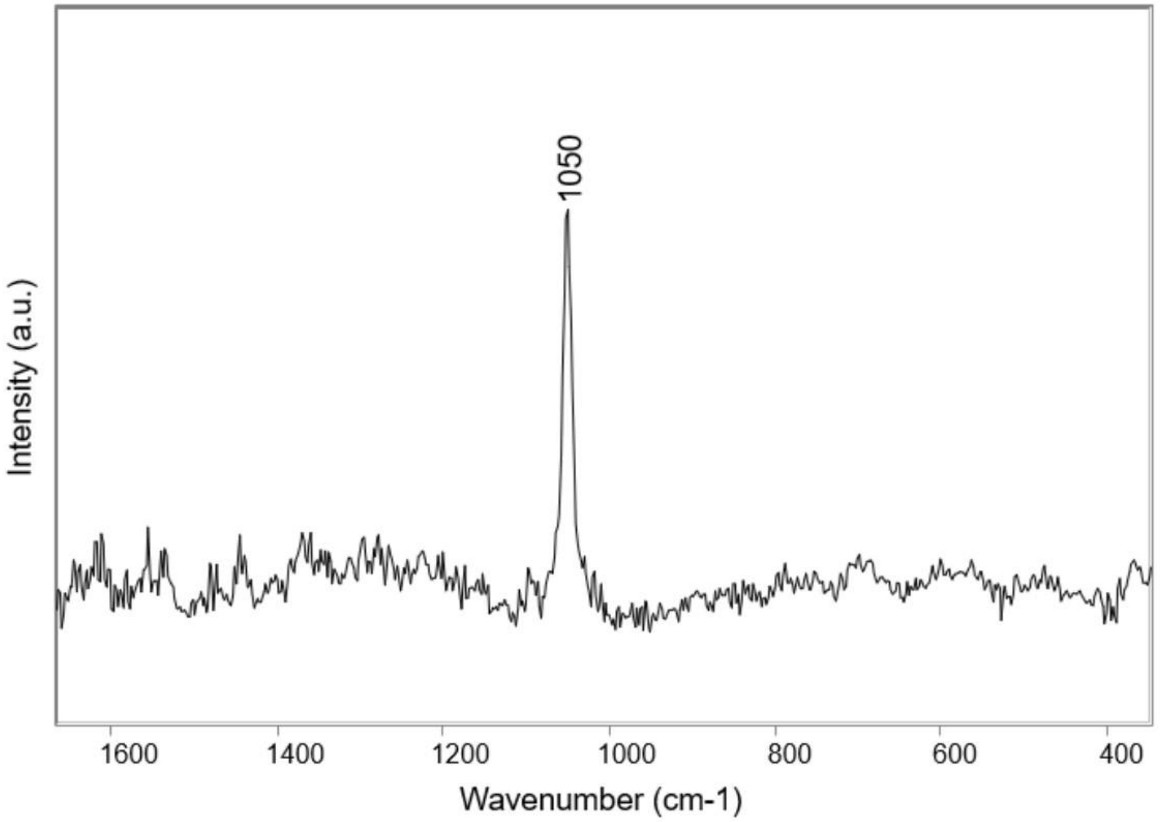

Figure S1. FT-Raman spectrum of the silver paint on the micro-sample taken from the dripping on the back of the "Concetto spaziale" near the cut.

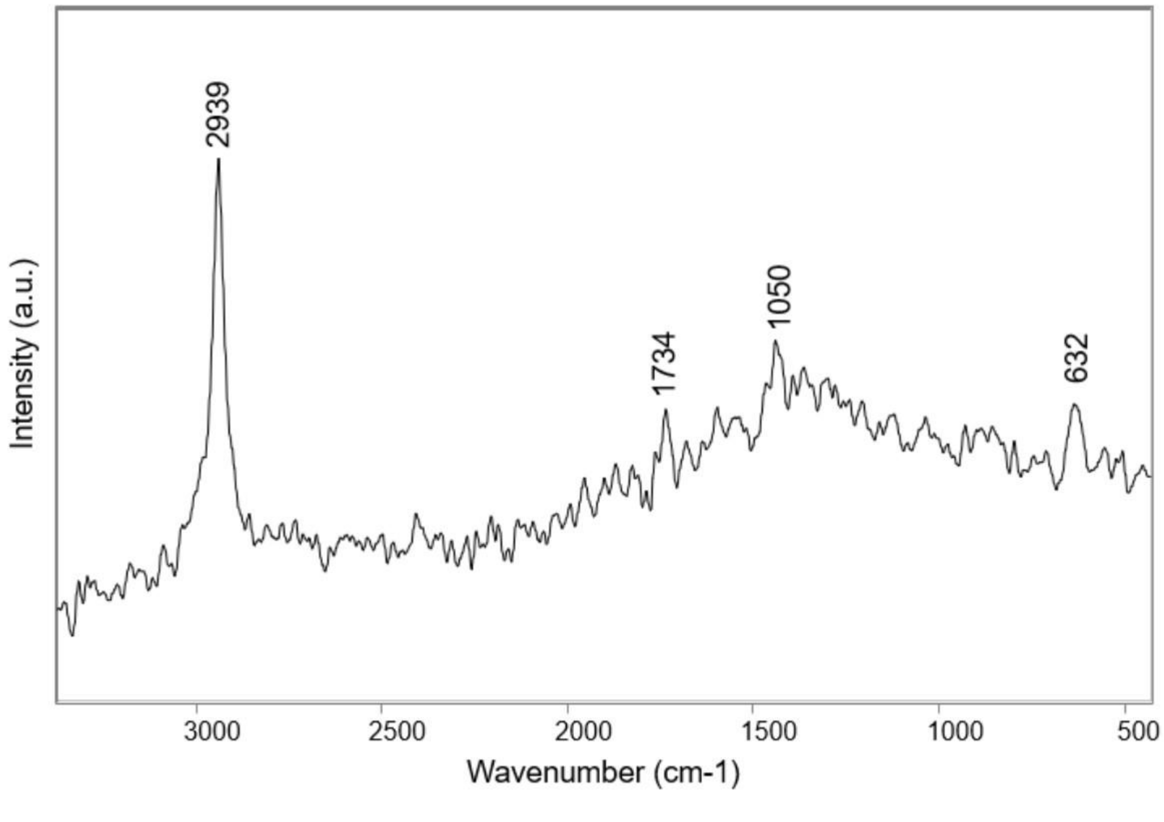

Figure S2. Raman spectrum obtained on the back of the canvas of the "Concetto spaziale" by Lucio Fontana.
